# 

**Published:** 1900-12

**Authors:** 


### Austin District Medical Association.

The Austin District Medical Society will meet in Austin in regular quarterly meeting the 20th of December. Annual election of officers and banquet. President, Dr. S. E. Hudson; Secretary, Dr. W. A. Harper.

### North Texas Medical Association.

The North Texas Medical Association will meet in Sherman on the 6th of December, inst. A large attendance is expected.

### South Texas District Medical Association.

The South Texas District Medical Association will meet in Houston December 12, 13 and 14 (inst.). A splendid program has been prepared. Excursion rates.

